# Plasma Type I IFN Protein Concentrations in Human Tuberculosis

**DOI:** 10.3389/fcimb.2019.00296

**Published:** 2019-08-22

**Authors:** Alba Llibre, Nicole Bilek, Vincent Bondet, Fatoumatta Darboe, Stanley Kimbung Mbandi, Adam Penn-Nicholson, Mark Hatherill, Flore Rozenberg, Thomas J. Scriba, Darragh Duffy

**Affiliations:** ^1^Laboratory of Dendritic Cell Immunobiology, Department of Immunology, Institut Pasteur, Paris, France; ^2^INSERM U1223, Institut Pasteur, Paris, France; ^3^South African Tuberculosis Vaccine Initiative, Division of Immunology, Department of Pathology, Institute of Infectious Disease and Molecular Medicine, University of Cape Town, Cape Town, South Africa; ^4^Université Paris Descartes & AP-HP, Groupe Hospitalier Universitaire Paris Centre, Service de Virologie, Paris, France

**Keywords:** tuberculosis, interferon, protein biomarker, Cytokines, IFNa, IFNb

## Abstract

Tuberculosis (TB) remains one of the leading causes of mortality worldwide, and a lack of understanding of basic disease pathogenesis is hampering development of new vaccines and treatments. Multiple studies have previously established a role for type I interferon (IFN) in TB disease. Type I IFNs are critical immune mediators for host responses to viral infection, yet their specific influence in bacterial infection remains unclear. As IFN-stimulated genes (ISGs) can have both stimulatory and inhibitory effects on immune function, clarifying the role of type I interferon in TB remains an important question. The quantification of interferon proteins in the circulation of patients has been restricted until the recent development of digital ELISA. To test the hypothesis that patients with active TB disease have elevated circulating type I IFN we quantified plasma IFNα and β proteins with Simoa digital ELISA in patients with active disease and asymptomatic *M. tuberculosis* infection. Strikingly no differences were observed between these two groups, while plasma from acute influenza infection revealed significantly higher plasma levels of both IFNα and IFNβ proteins. These results suggest a discordance between ISG mRNA expression by blood leukocytes and circulating type I IFN in TB.

## Introduction

Blood transcriptional signatures highly enriched for interferon (IFN)-stimulated genes (ISGs) are a hallmark of tuberculosis (TB) (Moreira-Teixeira et al., 2018), the leading cause of mortality from an infectious disease. Elevated expression of ISGs in blood leukocytes from patients with active TB disease has facilitated development of promising blood-based biomarkers that have diagnostic value for discrimination between individuals with TB disease and asymptomatic controls (Berry et al., 2010; Maertzdorf et al., 2011; Bloom et al., 2013; Kaforou et al., 2013; Zak et al., 2016). Consistent with this, we recently published an 11-gene blood signature primarily comprised of ISGs and showed that signature scores were consistently higher in TB cases than in controls with asymptomatic (latent) *Mycobacterium tuberculosis* (*M.tb*) infection (LTBI) (Darboe et al., 2018). These data, as well as studies of the immunobiology of TB suggest that type I and/or II IFNs trigger ISG expression in blood leukocytes, as observed in viral infections and a number of interferonopathies (McNab et al., 2015). Infection with *M.tb* is known to induce IFNγ-expressing T cell responses, which are necessary for immunological control of the bacterium to prevent disease progression (O'Garra et al., 2013), but it is currently not known if TB disease is associated with an elevated abundance of IFNα or IFNβ protein in peripheral blood. Although one study has previously reported no differences in circulating IFNα2 protein levels in TB disease (Berry et al., 2010), this conclusion relied on results from Luminex assays of which the majority of reported values were at the lower limit of detection of the assay, which we now know is insufficient for measuring physiological ranges of IFNα (Rodero et al., 2017).

We hypothesized that patients with active TB had elevated plasma levels of IFNα or β protein, as observed in respiratory viral infections. This hypothesis has not been previously tested as classical ELISAs lack the sensitivity required for reliable detection of IFNα or β in circulation. However, we recently utilized digital ELISA technology, based on counting individual enzyme-labeled immunocomplexes of proteins captured on paramagnetic beads in single-molecule arrays (Simoa), combined with unique high-affinity antibodies isolated from APS1/APECED mutation patients (Meyer et al., 2016) to detect plasma IFNα attomolar concentrations in viral infections, auto-immune disease, and interferonopathies (Rodero et al., 2017). Herein, we extended this approach to also measure IFNβ by digital ELISA, and using these novel assays we tested the hypothesis that type I IFN proteins are elevated in the blood during TB disease.

## Methods

### Patient Cohorts

Thirty patients (Active TB) with Xpert MTB/RIF (Cepheid) sputum-positive TB disease (HIV negative) and 30 QuantiFERON (QFT) Gold In-tube (Qiagen) positive asymptomatic adult controls (LTBI) were recruited from the Western Cape Province of South Africa, where TB is endemic (Table 1). Study participants provided written informed consent and the study protocol was reviewed and approved by the Human Research Ethics Committee of the University of Cape Town. As additional positive and negative controls for type I interferon responses we also included a cohort of French pediatric patients (11) with confirmed respiratory influenza viral infection and healthy donors (*n* = 30) from Paris (Table 1). Healthy donors (CoSImmGEn cohort of the Investigation Clinique et Accès aux Ressources Biologiques (ICAReB) platform, Center de Recherche Translationnelle, Institut Pasteur, Paris, France) and patients gave informed consent.

**Table 1:** Patient cohort characteristics.

**Subject type**	**Country**	**Numbers**	**Age (Median/range)**	**Sex (F/M)**
Healthy controls	France	30	46/24–67	6/24
Respiratory viral Infection	France	11	4/1–11	NA
Latent TB (LTBI)	South Africa	30	40.5/22–65	22/8
Active TB	South Africa	30	34/18–62	8/22

### Type I Interferon Assays

IFNα and IFNβ protein plasma concentrations were quantified by Simoa assays developed with Quanterix Homebrew kits as previously described (Rodero et al., 2017). For the IFNα assay, the 8H1 antibody clone was used as a capture antibody after coating on paramagnetic beads (0.3 mg/mL), the 12H5 clone was biotinylated (biotin/antibody ratio = 30/1) and used as the detector, and recombinant IFNα17 (Peprotech) used as the standard. For the IFNβ assay, the 710322-9 IgG1, kappa, mouse monoclonal antibody (PBL Assay Science) was used as a capture antibody after coating paramagnetic beads (0.3 mg/mL), the 710323-9 IgG1, kappa, mouse monoclonal antibody (PBL Assay Science) was biotinylated (biotin/antibody ratio = 40/1) and used as the detector antibody, and recombinant protein (PBL Assay Science) were used to quantify IFNβ concentrations. The limit of detection (LOD) of the IFNα and IFNβ assays were 0.5 fg/mL and 0.2 pg/mL, respectively. Functional activities of type I IFN were measured using an *in vitro* cytopathic assay that has been previously described (Lebon et al., 1979). Briefly IFN activity was determined by addition of patient plasma to Madin–Darby bovine kidney (MDBK) cells which were challenged with vesicular stomatitis virus to measure the viral cytopathic effect as compared with an IFN standard (Lebon et al., 1979). Blood ISGs were measured by an 11-gene blood signature score (ACS TB risk signature) by qRT-PCR from RNA isolated from PAXgene collected whole blood, as previously described (7). The raw data (Dataset_1) from the paper is available at Figshare, doi: 10.6084/m9.figshare.8799131.

### Statistical Analysis

**Figure 1:**
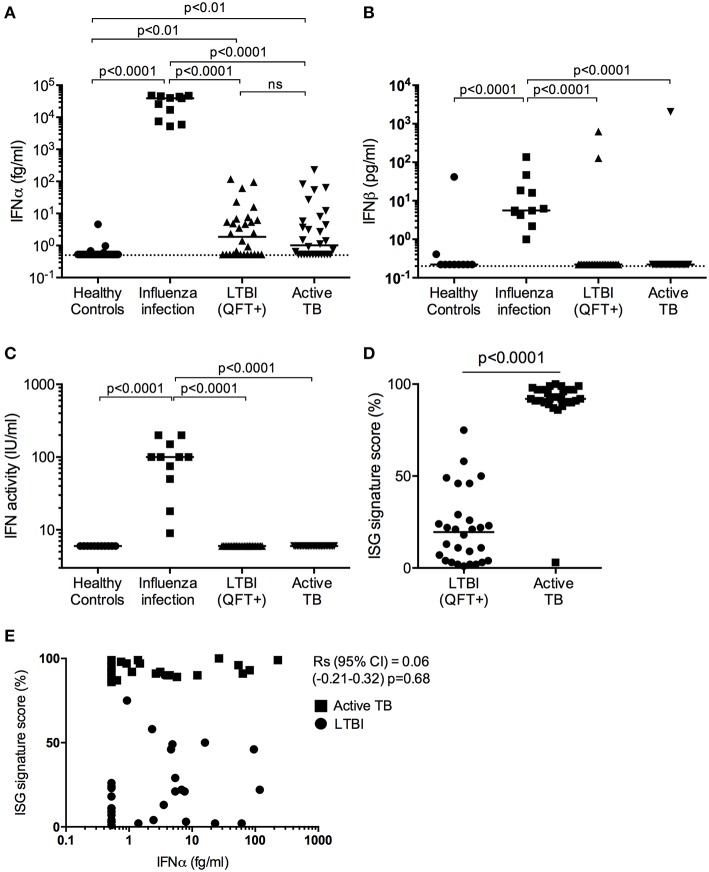
Type I IFN in active TB. **(A)** IFNα (fg/mL) and **(B)** IFNβ (pg/mL) concentrations and **(C)** IFN activity (IU/mL) in plasma from healthy controls (*n* = 30), influenza viral infection (*n* = 11), LTBI (*n* = 30) and active TB patients (*n* = 30). **(D)** ISG score as defined by an 11-gene signature (Darboe et al., 2018) in blood cells from LTBI (*n* = 30) and active TB patients (*n* = 30). (**A–C**: Kruskal-Wallis with Dunn's multiple comparison tests were performed; **(D)** Mann-Whitney *U*-test). (Digital ELISA limit of detection is indicated by the dotted line). **(E)** Spearman correlation between ISG score and plasma IFNα concentrations.

For multi-group comparisons, Kruskal-Wallis with Dunn's multiple comparison tests were performed (Figures 1A–C). For two-group comparisons, the Mann-Whitney U test was used (Figure 1D). Results are graphed on log scales due to the wide distributions of the data.

## Results

Plasma levels of IFNα were detectable in half of the TB patients and LTBI controls from South Africa, at levels below 100 fg/mL, but were not different between these two groups (Figure 1A). Although these South African donors had significantly (*p* < 0.01) higher plasma levels of IFNα protein (median 1–2 fg/mL) than healthy European controls, IFNα levels in individuals with influenza viral infection were orders of magnitude higher than individuals in the other three groups with a median concentration of 39 pg/mL (Figure 1A). Plasma levels of IFNβ, also measured by digital ELISA but with a commercially sourced antibody that gives lower sensitivity than the IFNα assay, were mostly undetectable. However, IFNβ was detected in all individuals with viral infection at a median concentration of 5 pg/mL, but was only detected in one TB patient and two LTBI controls from South Africa (Figure 1B). To further assess for potential type I interferons not detected by our Simoa assays, we tested for functional activities of type I IFN using an *in vitro* cytopathic assay that is less sensitive than the digital ELISA (Lebon et al., 1979). IFN activity in these plasma samples mirrored the observed protein level results; only individuals with viral infections had measurable bioactive type I IFN (Figure 1C). Finally, as shown previously and published by ourselves and others (Berry et al., 2010; Maertzdorf et al., 2011; Bloom et al., 2013; Kaforou et al., 2013; Zak et al., 2016; Darboe et al., 2018), active TB patients had significantly elevated 11-gene blood signature scores, based on ISG gene expression, as compared to LTBI (*p* < 0.0001; Figure 1D) (7). This ISG gene signature showed no correlation with plasma IFNα levels (Spearman correlation (95% CI) = 0.06 (−0.21 to 0.32), *p* = 0.68; Figure 1E).

## Discussion

Our data suggests a discordance between ISG mRNA expression by blood leukocytes and circulating type I IFN in TB, and reveals current limitations in our understanding of the immunobiology of *M.tb* infection. This capacity to directly measure type I IFN protein with unprecedented sensitivity can provide novel insights into the nature, regulation and consequences of the IFN response in TB disease. The virtual absence of IFNα and IFNβ in plasma suggests that type I IFN receptor (IFNAR) triggering in circulating immune cells to activate ISG expression must be happening in a different compartment, likely at the site of infection or in draining lymphoid tissues. Why this is different for TB as opposed to influenza viral infection, which revealed highly elevated IFN protein concentrations and previously described blood ISG signatures (Dunning et al., 2018), remains unclear. Alternatively, ISG expression may also be driven by IFNγ, which is well-recognized to be expressed by *M.tb*-specific T cells, unconventional T cells and natural killer cells during *M.tb* infection and TB disease (O'Garra et al., 2013). However, plasma levels of IFNγ and the abundance of IFNγ-expressing T cells do not allow discrimination between asymptomatic *M.tb* infection and TB disease (Pai, 2010), and therefore peripheral blood measures of type II IFN also cannot explain ISG mRNA expression by blood leukocytes. Since respiratory viral infections, such as influenza, are associated with high ISG mRNA expression (Dunning et al., 2018) and thus can lead to false positive transcriptomic TB signature results (Singhania et al., 2018), measuring type I IFN proteins could be used to differentiate between respiratory viral infections and TB. South African donors, latently infected with *M.tb* (defined based on a positive QFT response) were used as the most relevant control group to compare with active TB disease, and a healthy European population was also included. While it would have been interesting to also include a QFT negative South African population, the high prevalence of *M.tb* infection in adults makes this challenging, and the QFT+ individuals are healthy from a clinical standpoint. The differences in IFNα concentrations observed between the healthy European and LTBI controls are interesting, and may be due to either environmental or genetic differences, which will require further study in well-defined population cohorts.

Furthermore, identification of the origin and source of type I interferon will require additional studies in relevant animal models, where access to infected tissue is possible. Additionally given the transient nature of type I IFN protein secretion, immune stimulation may be required to reveal such potential differences. This will help to clarify the controversial role of type I interferons in TB disease and may help in the future development of new diagnostic tools, vaccines and treatments.

## Data Availability

All datasets generated for this study are available at Figshare, doi: 10.6084/m9.figshare.8799131.

## Ethics Statement

Thirty patients (Active TB) with Xpert MTB/RIF (Cepheid) sputum-positive TB disease (HIV negative) and 30 QuantiFERON (QFT) Gold In-tube (Qiagen) positive asymptomatic adult controls (LTBI) were recruited from the Western Cape Province of South Africa, where TB is endemic (Table 1). Study participants provided written informed consent and the study protocol was reviewed and approved by the Human Research Ethics Committee of the University of Cape Town. As additional positive and negative controls for type I interferon responses we also included a cohort of French pediatric patients (11) with confirmed respiratory influenza viral infection and healthy donors (30) from Paris (Table 1). Healthy donors (CoSImmGEn cohort of the Investigation Clinique et Accès aux Ressources Biologiques (ICAReB) platform, Center de Recherche Translationnelle, Institut Pasteur, Paris, France) and patients gave informed consent.

## Author Contributions

DD and TS designed the study and wrote the manuscript. AL, NB, VB, FD, SM, AP-N, MH, and FR generated and analyzed data. All authors contributed to manuscript revision, read and approved the submitted version.

### Conflict of Interest Statement

The authors declare that the research was conducted in the absence of any commercial or financial relationships that could be construed as a potential conflict of interest.
